# Platelet–Neutrophil Association in NETs-Rich Areas in the Retrieved AIS Patient Thrombi

**DOI:** 10.3390/ijms232214477

**Published:** 2022-11-21

**Authors:** Ghulam Jeelani Pir, Aijaz Parray, Raheem Ayadathil, Sajitha V. Pananchikkal, Fayaz Ahmad Mir, Islam Muhammad, Ahmed Abubakar, Nueman Amir, Sohail Hussain, Khawaja H. Haroon, Ahmad Muhammad, Yahya Imam, Satya Narayana Patro, Naveed Akhtar, Aymen Zakaria, Saadat Kamran

**Affiliations:** 1 The Neuroscience Institute and Academic Health System, Hamad Medical Corporation, Doha 3050, Qatar; 2 Qatar Metabolic Institute, Academic Health System, Hamad Medical Corporation, Doha 3050, Qatar; 3 College of Medicine, Qatar University (QU) Health, Qatar University, Doha 2713, Qatar; 4 Max Rady College of Medicine, Rady Faculty of Health Sciences, University of Manitoba, Health Sciences Centre Sherbrook Street, Winnipeg, MB R3A 1R9, Canada; 5 Department of Radiology, UAMS Medical Center, 4301 W. Markham St., Little Rock, AR 72205, USA; 6 Weill Cornell Medical School, Doha 24144, Qatar

**Keywords:** AIS, fibrin, HMGB1, leukocytes, NETs, neutrophils, stroke, t-PA, von Willebrand factor, platelets

## Abstract

Histological structure of thrombi is a strong determinant of the outcome of vascular recanalization therapy, the only treatment option for acute ischemic stroke (AIS) patients. A total of 21 AIS patients from this study after undergoing non-enhanced CT scan and multimodal MRI were treated with mechanical stent-based and manual aspiration thrombectomy, and thromboembolic retrieved from a cerebral artery. Complementary histopathological and imaging analyses were performed to understand their composition with a specific focus on fibrin, von Willebrand factor, and neutrophil extracellular traps (NETs). Though distinct RBC-rich and platelet-rich areas were found, AIS patient thrombi were overwhelmingly platelet-rich, with 90% of thrombi containing <40% total RBC-rich contents (1.5 to 37%). Structurally, RBC-rich areas were simple, consisting of tightly packed RBCs in thin fibrin meshwork with sparsely populated nucleated cells and lacked any substantial von Willebrand factor (VWF). Platelet-rich areas were structurally more complex with thick fibrin meshwork associated with VWF. Plenty of leukocytes populated the platelet-rich areas, particularly in the periphery and border areas between platelet-rich and RBC-rich areas. Platelet-rich areas showed abundant activated neutrophils (myeloperoxidase^+^ and neutrophil-elastase^+^) containing citrullinated histone-decorated DNA. Citrullinated histone-decorated DNA also accumulated extracellularly, pointing to NETosis by the activated neutrophils. Notably, NETs-containing areas showed strong reactivity to VWF, platelets, and high-mobility group box 1 (HMGB1), signifying a close interplay between these components.

## 1. Introduction

Rapid recanalization of the occluded vessel is the mainstay of contemporary acute stroke treatment. The treatment of acute stroke is limited to pharmacological thrombolysis within 4.5 h of stroke onset with or without thrombectomy and thrombectomy alone up to 24 h [1]. Because of time limitations, thrombolysis is available to less than 15% of patients. Moreover, in patients who receive rt-PA, more than half fail to recanalize. The mechanisms of reperfusion failure are complex and not fully understood. This includes but is not limited to time from stroke onset to thrombolysis, thrombolytic dose, clot size, clot distance, clot permeability, collateral circulation, and clot composition [2,3].

Two main components of thrombi are RBCs and platelets. Based on the abundance of these two components, thrombi can be classified into three main types, RBC-rich, mixed, and platelet-rich. Among these, platelet-rich clots have been reported to be more resistant to thrombolysis by t-PA [4,5,6]. The precise role of RBCs in thrombosis remains largely unknown and they have rather been regarded as passive bystanders. Platelets (alongside coagulation factors and endothelial cells) on the other hand are central to thrombosis [7]. Platelet tethering that initiates thrombus formation is aided by ligands such as the large multimeric plasma protein, von Willebrand factor [8], and thrombospondin-1 [9,10]. This leads to a chain of events that result in platelet activation mostly by the exposed collagen [11], adhesion, and aggregation. In the following events that culminate in platelet plug formation, platelets spread and release their granular contents including von Willebrand factor which in turn leads to more platelet recruitment aided by the glycoprotein Ib platelet-subunit alpha GPIbα (CD42b) on the incoming platelets [12,13,14]. Fibrin and von Willebrand factor scaffold and further stabilize the thrombus plug [15,16]. Additionally, NETs have been recently reported in retrieved thrombi from AIS patients [17,18]. NETs, first described in 2004 by Brinkmann et al., are extracellular filamentous DNA structures comprising histones and antimicrobial proteins. Primarily, NETs are known to be involved in immunity [19,20,21,22]. In response to bacterial infection and/or inflammation, NETs released by granulocytes serve as adhesive support to trap and limit bacterial dissemination in the body [19,20,21,22]. The likelihood of VWF and NETs providing additional scaffolding and strength comes from studies where VWF digestion by ADAMTS13 decreased thrombus formation in mice [23,24,25,26] or DNase 1 treatment induced acceleration of t-PA mediated ex vivo thrombolysis of retrieved thrombi [17,18]. NET formation by granulocytes in turn was recently reported to be mediated and driven by an interplay between platelets and neutrophils aided by platelet-specific high-mobility group box 1 (HMGB1) [27].

The risk factor and clinical profiles of stroke patients may differ from area to area across the world, influencing the global variations in etiology, occurrence, and outcome [28]. Countries in the Southeast Asian and Eastern Mediterranean regions of the World Health Organization contribute nearly 60% of the global cardiovascular disease burden and up to 40% of global stroke deaths [29,30]. The stroke-related risk factors relatively specific to Asia include stroke at a younger age, premature atherosclerosis, higher prevalence of intracranial atherosclerosis, pre-diabetes, and new-onset diabetes mellitus (DM), a higher prevalence, predisposition to, and more extensive Coronary Artery Disease (CAD) at a younger age compared with Whites [31,32,33,34].

To our knowledge, there is a paucity of data regarding clot composition from acute stroke patients from Asia. The objective of this study, therefore, was to report the structural organization of different components in the retrieved clots from acute stroke patients from the multiethnic Asian and North African cohorts.

## 2. Results

### 2.1. Clinical Charecteristics of the AIS Patients

**Figure 1:**
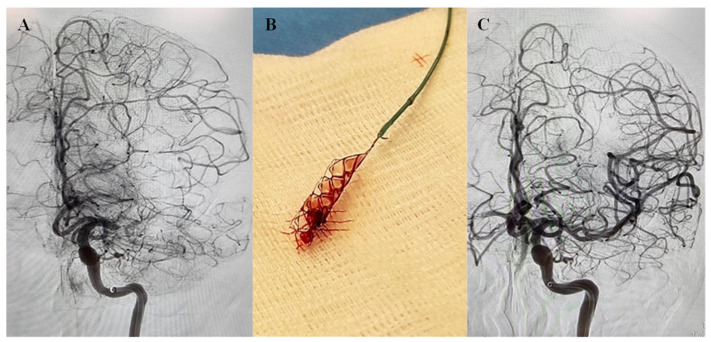
Cerebral Angiogram of acute stroke with left middle cerebral artery (MCA) occlusion. (**A**) Cerebral angiograph showing left MCA M1 occlusion. (**B**) After two passes with Solitaire (4 × 20 mm), vessel recanalizes (TICI 3). (**C**) Post-thrombectomy angiogram showing complete recanalization of the left MCA.

The clots were obtained from twenty-one consecutive acute stroke patients with large vessel occlusion that underwent thrombectomy between July 2019 and January 2021 (Figure 1). There were 17 males and 4 females, with a mean age of 50.73 ± 11.04 years. Stroke risk factors were diabetes: 47.6%; hypertension: 52.4%; dyslipidemia: 28.6%; smoking: 23.8%; and atrial fibrillation: 14.2%. Before thrombectomy, two patients were taking antiplatelets and one anticoagulant. As per TOAST classification [35], 47.6% presented with large artery disease, 38% cardioembolic, and 14.2% embolic stroke of undetermined source (ESUS). Ten patients were provided thrombolysis followed by thrombectomy while 11 patients underwent thrombectomy without thrombolysis. The thrombi were retrieved from the following vessels: middle cerebral artery occlusion (MCA) (n = 19), and posterior cerebral artery (PCA) (n = 2) patients. The door to recanalization time was 184 min (range 111–326). Supplementary Table S1 summarizes the further clinical characteristics of the AIS patients.

### 2.2. AIS Patient Thrombi Contain Distinct Patterns of Areas Rich in RBCs and Platelets

**Figure 2:**
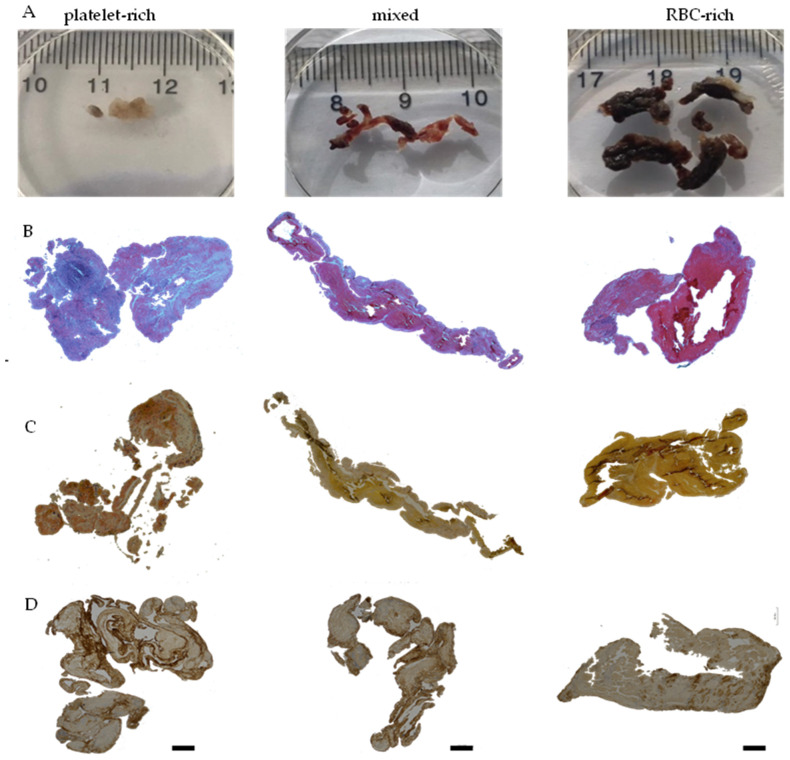
Distinct red blood cell (RBC)-rich and platelet-rich areas are seen in stroke thrombi. (**A**) Representative photomacrographs of the platelet-rich, mixed, and RBC-rich thrombi were retrieved from ischemic stroke patients. (**B**) Martius Scarlet Blue (MSB) staining of the three representative thrombi sections. Fibrin-rich areas appear red while RBC-rich areas appear yellow. (**C**) Hematoxylin and Eosin (H and E) staining depicting RBC-rich areas (red) and RBC-poor areas (light pink). (**D**) Immunohistochemical staining shows von Willebrand factor (VWF) in the three representative thrombi sections. Scale bars (**B**–**D**) = 500 μm.

All the AIS patient thrombi retrieved by endovascular thrombectomy were sectioned and stained with classical hematoxylin and eosin (H&E) and Martius Scarlet Blue (MSB) to identify their structural components. Macroscopically, thrombi were heterogenous in size, shape, and color (Figure 2A–C, Supplementary Figure S1A–F).

Histological examination with H&E and MSB showed a considerable difference in the overall appearance of thrombi. Two distinctive patterns of thrombus areas were identified: (i) areas rich in RBCs/poor in fibrin and platelets, stained red on H&E and yellow on MSB (designated by letter R), and (ii) areas poor in RBCs/rich in fibrin and platelets, appearing light pink on H&E and pink to red on MSB staining (designated by letter P). Immunohistochemical examination specific to Von Willebrand factor and blood platelets showed intense staining in discrete areas rich in fibrin in all the AIS patient thrombi (Figure 2D, Supplementary Figure S1G).

**Figure 3:**
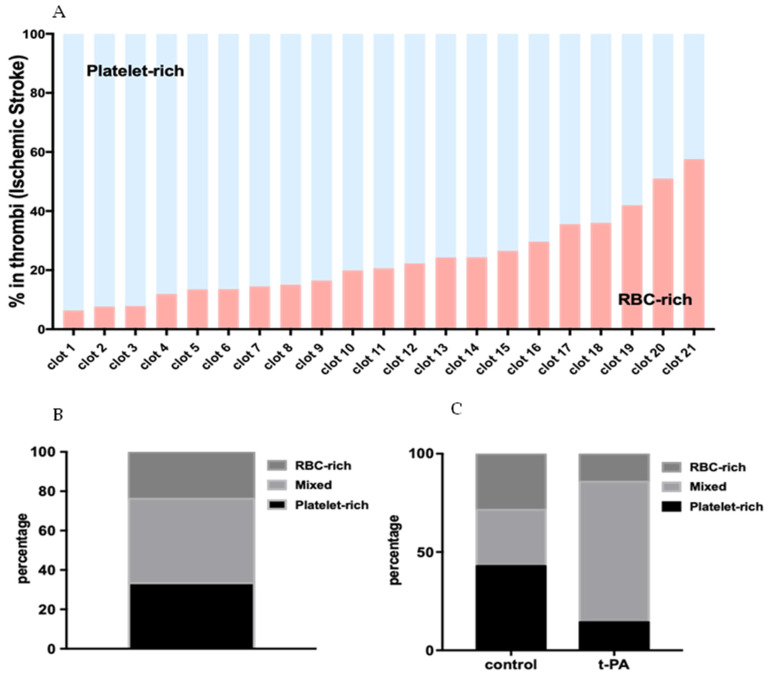
Composition of the stroke thrombi. (**A**) Quantitative analysis of the stroke thrombi using H&E staining was performed and the percentage of RBC-rich areas (red) and platelet-rich areas (light blue) was determined. All the thrombi contain significant amounts of platelet-rich and RBC-rich areas. Individual thrombi differ in composition with some showing an abundance of platelet-rich areas while others an abundance of RBC-rich areas (n = 21, vertical bars). (**B**) Bar diagram showing the classification of stroke thrombi based on the percentage of platelet-rich and RBC-rich areas. The majority consisted of mixed thrombi. (**C**) Percentage of RBC-rich, mixed, and platelet-rich thrombi in control vs. group that received t-PA.

Based on the abundance of RBC-rich areas, thrombi were categorized as RBC-rich/platelet-poor, mixed, and RBC-poor/platelet-rich (Figure 3A). Quantification of thrombus constituents from individual sections of each thrombus revealed that thrombi contained RBC-rich contents ranging from 6.36 ± 0.2% to 57.56 ± 7.4%, and the corresponding mean platelet-rich contents from 93.64 ± 0.2% to 42.44 ± 7.4% (Figure 3A). As per this classification, 33.33% of the thrombi in this study turned out to be platelet-rich, 42.86% mixed, and 23.81% RBC-rich (Figure 3B).

AIS patients who received tPA, in general, had a higher percentage of mixed thrombi (71.42%) compared with the group that did not (control: 28.57%), and a lower percentage of platelet-rich and RBC-rich (14.28% each) thrombi compared with the group that did not (control: platelet-rich: 42%; RBC-rich: 28.57%) (Figure 3C).

### 2.3. RBC-Rich Areas Show Heavily Populated RBCs Packed in Thin Fibrin Network

**Figure 4:**
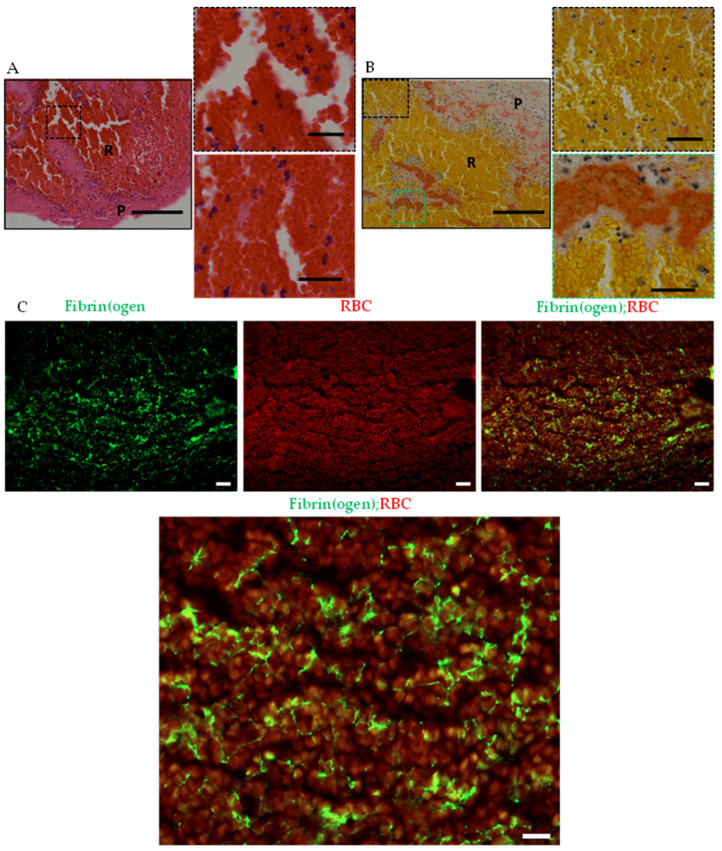
Densely packed RBC in fibrin meshwork present in RBC-rich areas (R). (**A**) Hematoxylin and Eosin (H&E) staining of stroke thrombi at low (left panel) and high (right panels) magnifications show a high abundance of RBC (appearing red) surrounded by fibrin (pink) with sparsely populated or no nucleated cells (blue) in RBC-rich areas. Scale bars: 200 μm, left panel; 25 μm, right panels (**B**) Martius Scarlet Blue (MSB) staining of stroke thrombi at low (left panel) and high (right panels) magnifications show densely populated RBCs (appearing yellow) in thin Fibrin network (appearing red). Sparsely populated or no nucleated cells (blue) are seen in the RBC-rich areas. Scale bars: 200 μm, left panel; 25 μm, right panels (**C**). Immunofluorescence staining of stroke thrombi. Fibrinogen-specific antibody was used to visualize fibrin (first panel, green) and RBCs by autofluorescence (mid-panel, red). Fibrin is interwoven in densely populated RBCs (right panel, low magnification; and lower panel, high magnification). Scale bars are 30 μm. P: platelet-rich area; R: RBC-rich area.

To obtain more insights into the microscopic details of thrombi contents in different areas, we first investigated the RBC-rich areas using H&E and MSB stainings at higher magnifications. On H&E and MSB stainings, RBC-rich areas showed crowded RBCs with sparsely populated or no nucleated cells (Figure 4A,B). Thick fibrin strands (pink in Figure 4A, red in Figure 4B) were seen towards the periphery of the RBC-rich areas while thin network of fibrin strands could be seen penetrating the crowded RBCs on MSB staining (Figure 4B).

To corroborate these results, we performed immunofluorescence imaging of the thrombi sections. Co-immunofluorescence using antibody specific to fibrin(ogen)and autofluorescence emitted by RBCs at 555 nm, showed a network of thin fibrin strands scattered in tightly packed RBCs (Figure 4C). Von Willebrand factor (VWF), a large multimeric plasma glycoprotein, plays a key role in thrombus formation by recruiting platelets, and strengthening the resulting platelet plug together with fibrin(ogen) [36,37]. In our stainings, however, very little VWF and platelets were detected in the RBC-rich areas (Supplementary Figure S2). Taken together, tightly packed RBCs in a thin fibrin network characterize the RBC-rich areas in ischemic stroke thrombi.

### 2.4. Dense Fibrin Network Associated with VWF and Infiltrated Platelets Define Platelet-Rich Areas

**Figure 5:**
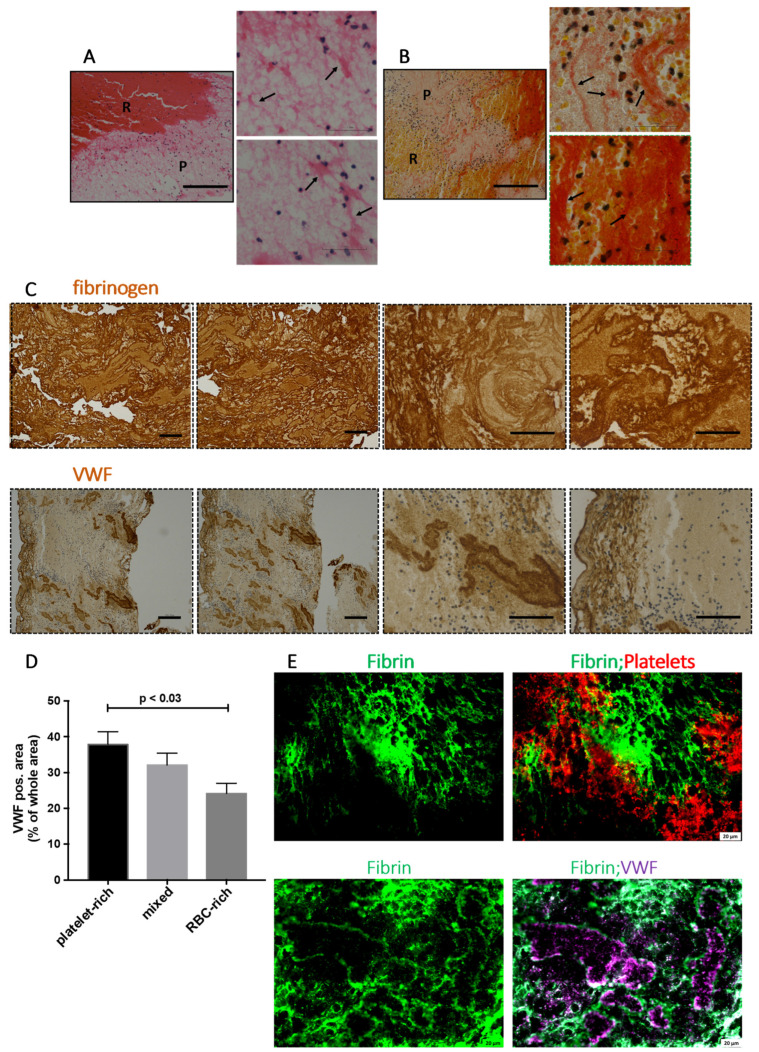
Platelet-rich areas show infiltrated platelets in dense fibrin networks lined with von Willebrand Factor (VWF). (**A**) Hematoxylin and Eosin (H&E) staining of AIS patient thrombi at low (left panel) and high (right panels) magnifications show profuse fibrin network (appearing light pink, black arrows) with heavily populated nucleated cells (blue) in platelet-rich (P) areas. Scale bars = 200 μm. (**B**) Martius Scarlet Blue (MSB) staining of stroke thrombi at low (left panel) and high (right panels) magnifications show dense fibrin network (appearing red) in platelet-rich (P) areas. More nucleated cells (blue) are seen in the platelet-rich (P) areas. Scale bars = 200 μm. (**C**) Immunohistochemical staining of retrieved stroke thrombi using antibodies specific to fibrin(ogen) (top panel) and VWF (bottom panel). Higher magnifications of the representative stainings are shown on the right two panels each. Outer edges show dense VWF staining whereas inside areas show discrete staining. Scale bars = 200 μm on the left four panels and 100 μm in right four panels. (**D**) Bar diagram showing the percentage of total VWF^+^ area in all three thrombi subgroups (platelet-rich, mixed, and RBC-rich). The data points represent mean ± SEM of the percentages. Two-tailed *t*-test (**E**) Immunofluorescence staining of stroke thrombi was used to visualize fibrin(ogen) (green), platelets (CD42b, red), and VWF (purple). A profuse fibrin network lined with VWF and infiltrated by platelets (white arrows) is seen. Scale bars are 20 μm. P: platelet-rich area; R: RBC-rich area.

Next, we set out to investigate the platelet-rich areas. On H&E stainings, platelet-rich areas appeared light pink. In sharp contrast to the RBC-rich areas (see Figure 4B), platelet-rich areas contained densely populated nucleated cells (blue) as seen on H&E and MSB staining (Figure 5A,B). On MSB staining, platelet-rich areas showed dense fibrin material (red) visible throughout, with some areas rich in a highly condensed fibrin network. These results were corroborated by immunohistochemical staining using an antibody specific to fibrinogen, showing a strong fibrin staining in all the AIS patient thrombi (Figure 5C, fibrinogen, top panel). Similarly, VWF immunohistochemical staining showed discrete VWF-positive areas in all AIS patient thrombi mostly on the outer edges and in platelet-rich areas (Figure 5C, VWF, bottom panel). Since the AIS patient thrombi samples in this study were platelet-rich and the platelet-rich areas in general showed strong VWF staining, we compared the three thrombi classes (platelet-rich, mixed, and RBC-rich thrombi) in their overall VWF content. Indeed, RBC-rich thrombi showed the least total VWF content compared with the platelet-rich and mixed thrombi (Figure 5D).

To gain insights into the arrangement of these components in the retrieved thrombi, we performed immunofluorescence imaging of thrombi sections using antibody combinations specific to fibrin(ogen) and platelets or fibrin(ogen) and VWF. Discrete zones demarcated by dense fibrin network (green) with infiltrated platelets (red) and lined by VWF (purple) were apparent on co-immunofluorescence images (Figure 5E). This is in sharp contrast with RBC-rich areas, where densely packed RBCs were found in thin fibrin network without any substantial VWF (see Supplementary Figure S2_upper panel). Taken together, the presence of VWF in a dense fibrin network in platelet-rich areas shows that the two components work together to scaffold and stabilize the thrombi.

### 2.5. Leukocytes Majorly Populate the Platelet-Rich Areas in AIS Patient Thrombi

**Figure 6:**
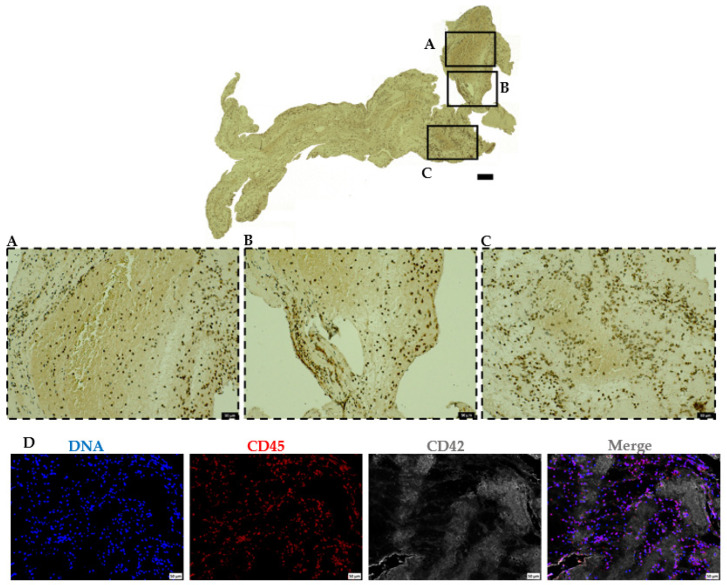
Abundant leukocyte accumulation mainly at the interface between platelet-rich and RBC-rich areas. (**A**–**C**) Immunohistochemical staining of retrieved AIS patient thrombi to visualize leukocytes using CD45 antibody (upper panel). Higher magnification of the representative staining is shown in inserts (lower panels). Scale bars: 200 μm (top panel) and 50 μm (lower panels). (**D**) Representative ischemic stroke patient thrombi stained for leukocytes (CD45, red), platelets (CD42b, grey), and DNA (DAPI, blue). Scale bars: 50 μm.

In addition to the classical components of thrombi (platelets, fibrin, and red blood cells), H&E and MSB stainings showed highly populated polymorphonuclear cells (blue) (see Figure 4A,B and Figure 5A,B). To characterize this cellular population, we used CD45 antibody, a marker specific to leukocytes. Immunohistochemical staining showed the presence of nucleated CD45 positive cells (leukocytes) in all the thrombi sections. While the leukocyte populations were mainly found in the outer edges of platelet-rich areas, and at the RBC-rich and platelet-rich interface, the RBC-rich areas were sparsely populated but not completely devoid of the leukocyte populations (Figure 6, Supplementary Figure S3A,B). Furthermore, immunofluorescence staining with CD45 antibody and DAPI (DNA) demonstrated that most nucleated cells in IS patient thrombi are indeed leukocytes (Figure 6D, Supplementary Figure S3C).

### 2.6. AIS Patient Thrombi Show Neutrophil Extracellular Traps (NETs) in the Platelet-Rich Areas and at the Interface between RBC-Rich and Platelet-Rich Areas

**Figure 7:**
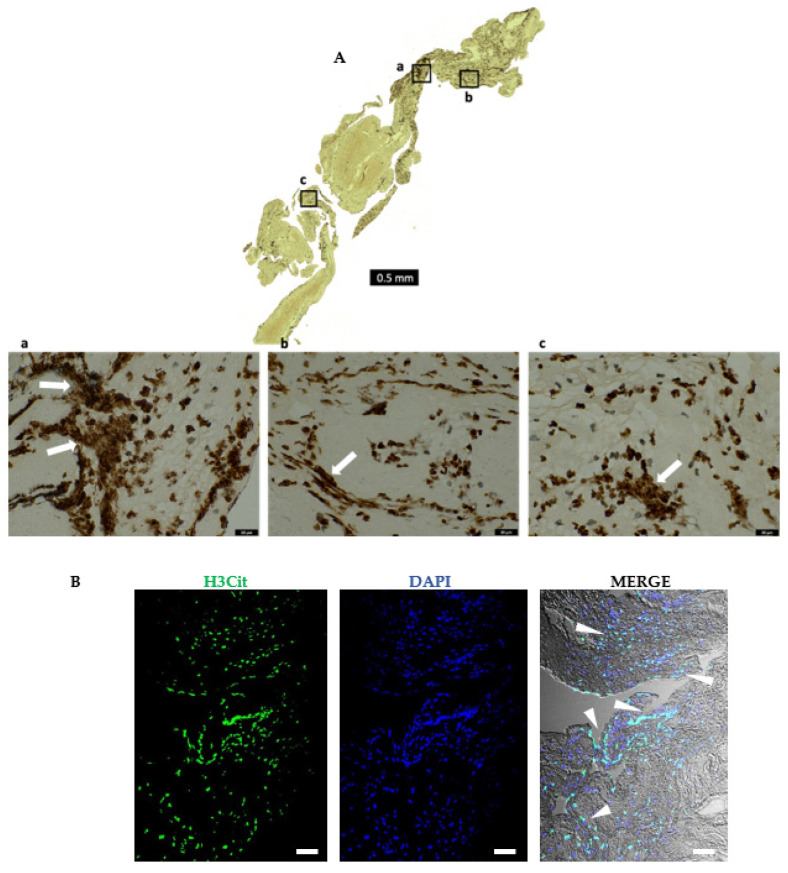
AIS patient thrombi show the constitutive presence of NETs. (**A**) Representative immunohistochemical staining of retrieved stroke thrombus using an antibody against citrullinated histone (H3Cit), a marker for NETs. Abundant NET formation that appears intracellular as well as diffused extracellularly (white arrows in (**a**–**c**)), mainly in the platelet-rich areas and at the thrombi periphery can be seen. Scale bars: 500 μm: upper panel, and 20 μm: bottom panels. (**B**) Representative immunofluorescence image of retrieved stroke thrombus stained for DNA (DAPI, blue), and with antibody against citrullinated histone (H3Cit, green). The diffused nuclear material mainly in the periphery (blue in DAPI) colocalizes with H3Cit (green), as shown by arrowheads in the merged image. Scale bars are 100 μm.

A careful observation of the H&E and histochemical staining showed diffused nuclear material that appeared extracellular and located more specifically towards the outer layers of all the thrombi (Supplementary Figure S4A,B). The diffused extracellular nature of this nucleic acid material pointed to NET formation. We confirmed this by immunostaining using an antibody specific to citrullinated histones (H3Cit), a known marker of NET formation, and observed a profuse nuclear as well as extra-cellular staining (Figure 7).

**Figure 8:**
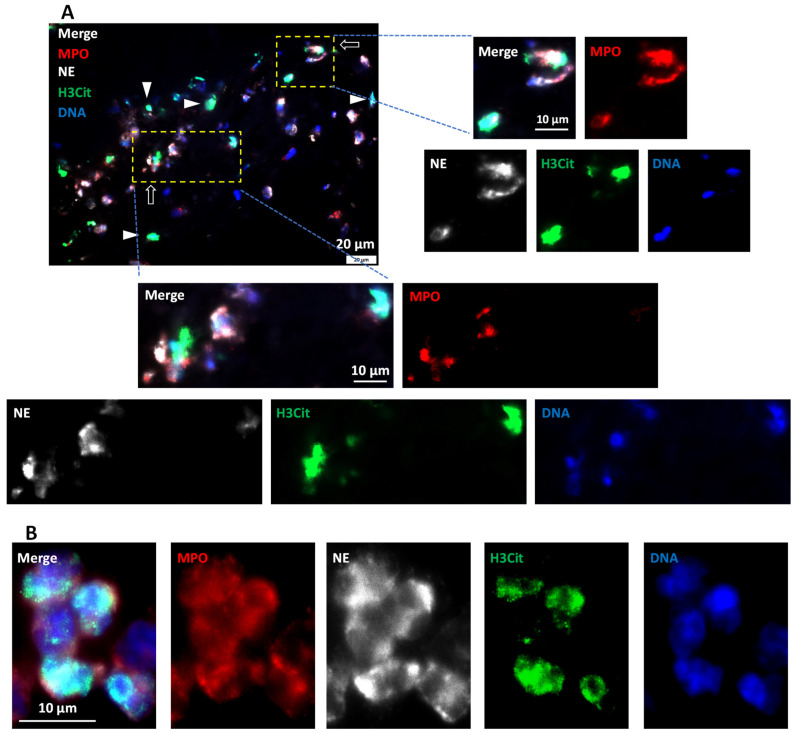
AIS patient thrombi show NET formation in platelet-rich areas. (**A**) Representative immunofluorescent image of ischemic stroke patient thrombi stained for DNA (blue), and with antibodies against neutrophils (MPO, red, and NE, white) and citrullinated histone (H3Cit, green). Neutrophils are seen at different NET-forming stages (white arrows). Moreover, visible are extracellular NETs (white arrowheads) depicting complete NETosis. Scale bar: A: 20 μm and insets: 10 μm. (**B**) Representative ischemic stroke patient thrombi at higher magnification show NET-forming neutrophils with nuclear decondensation and marked with intracellular H3Cit staining. Scale bar: 10 μm.

To determine the nature and source of this citrullinated histone-decorated nuclear material, immunocytochemistry using NET-marker H3Cit and neutrophil-specific markers neutrophil elastase, (NE) and myeloperoxidase (MPO) was performed; since neutrophils are the most abundant leukocytes and are specifically involved in NET formation [38]. Abundant neutrophils populated all the thrombi as seen by NE and MPO co-staining (Supplementary Figure S4C,D). Among these neutrophils (cells co-positive for NE and MPO), numerous stained positive for H3Cit, indicating NET-forming cells at different stages of NETosis (Figure 8A,B). Moreover, extracellular NETs (co-positive for H3Cit and DAPI) were found in all the AIS patient thrombi (Figure 8 inset, Supplementary Figure S5), indicating extracellular nuclear material released by neutrophils after complete NETosis. Taken together, all the retrieved thrombi showed abundant NET formation that derived their origin from neutrophils.

### 2.7. Areas Rich in NETs Show Close Association of Platelets and Neutrophils in Thrombi

**Figure 9:**
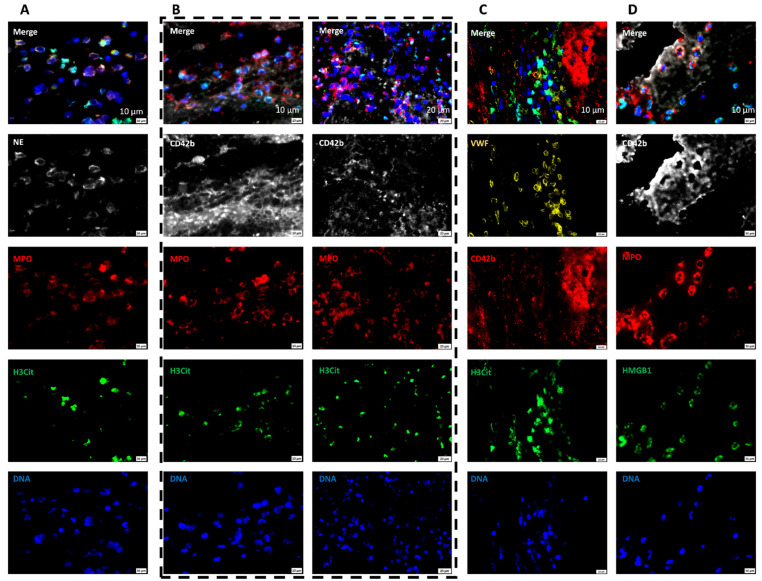
Platelet-rich areas show a close association between neutrophil-specific and platelet-specific components. (**A**) Representative ischemic stroke patient thrombi stained for neutrophils (MPO, red and NE, white), NETs (H3Cit, green), and DNA (DAPI, blue). Scale bars: 10 μm. (**B**) Two representative ischemic stroke patient thrombi stained for platelets (CD42b, white), neutrophils (MPO, red), NETs (H3Cit, green), and DNA (DAPI, blue). Platelets are seen in close association with NETs. Scale bars: 10 μm, left panel; 20 μm, right panel. (**C**) Representative ischemic stroke patient thrombi stained for von Willebrand factor (VWF, violet), platelets (CD42b, red), NETs (H3Cit, green), and DNA (DAPI, blue). Von Willebrand factor is seen at the interface with platelets in NET-rich areas. Scale bars: 10 μm (**D**) Representative ischemic stroke patient thrombi stained for platelets (CD42b, white), HMGB1 (green), neutrophils (MPO, red), and DNA (DAPI, blue). HMGB1 is visible interspersed between platelets and neutrophils. Scale bars: 10 μm.

In our observation, NETs were detected mainly in the platelet-rich areas, areas bordering RBC-rich and platelet-rich areas and at the periphery. To reveal the structural organization of platelet- and neutrophil-derived components in these areas, we performed triple staining of thrombi using DAPI (DNA), and antibodies against citrullinated histones (H3Cit–NETs), neutrophils (MPO), platelets (CD42b), and VWF. Aggregates of platelets were found interspersed with neutrophils, majority of which also co-stained for NETs (Figure 9A,B). Likewise, VWF, involved in the initial platelet activation, was found furrowing the platelet-aggregates that were interspersed with NETs (Figure 9C). Another molecule that is known to aid in platelet–neutrophil interaction which culminates in NET formation is platelet-derived HMGB1 [27,39,40]. We therefore looked at the arrangement of HMGB1 in the retrieved AIS patient thrombi. HMGB1 was found mainly localized at the interface bridging the platelets and NETs-filled neutrophils in the AIS patient thrombi (Figure 9D). In conclusion, the individual components show an arrangement where VWF, platelets, and NETs within and outside neutrophils are closely packed together and are likely interacting during the thrombus formation.

## 3. Discussion

The main finding of this study is the identification of platelet-rich thrombi abundant in fibrin, VWF, and NETs retrieved from AIS patients in this geographical region, a plausible explanation behind a high t-PA resistance shown by the patients during initial attempts of pharmacological recanalization.

Retrieved thrombi were heterogenous in shape and histology, and consisted of two distinct areas: one, rich in RBCs and poor in other cellular populations; and two, rich in platelets and other cellular populations. The two areas differed in their structural composition and the arrangement of the individual components, with some areas showing an abundance of only fibrin while others showing an abundance of all these components similar to the earlier studies [18,24,41,42,43,44,45,46]. RBC-rich areas contained only thin fibrin network scattered in trapped RBCs without any substantial von Willebrand factor. In contrast, platelet-rich areas showed dense fibrin network interwoven with von Willebrand factor and densely populated leukocytes, majority of which were activated neutrophils. These activated neutrophils were found at different stages of NETosis, some were filled with citrullinated histone-decorated decondensed chromatin while others released these contents into the extracellular space. Areas rich in NETs showed close association of components known to activate platelets viz a viz VWF, and trigger NETosis in neutrophils viz a viz high-mobility group box 1 (HMGB1).

Despite fibrin, VWF, platelets, nucleated cells, RBCs, and NETs being the common ingredients, AIS thrombi shape and histology heterogeneity reflect the varying clinical patient features that govern the circulation dynamics such as blood flow, shear, and turbulence conditions at the thrombus site. Of note, the nucleated cells (the majority of which turned out to be leukocytes upon immunochemical examination) were more abundant at the border areas between platelet-rich and RBC-rich areas. This structural pattern was more apparent with MSB-staining, where there was a presence of a dense fibrin network and nucleated cells mostly in the platelet-rich areas, and rather structurally simple RBC-rich areas. The role of platelets in thrombosis is well-established. Together with coagulation factors and endothelial cells, platelets primarily contribute to blood coagulation and thrombin generation. RBCs, generally regarded as passive bystanders, might play a greater role in thrombosis as shown by the recent clinical evidence. For example, RBCs may contribute to thrombosis through multimodal effects such as their ability to: alter blood viscosity and interact directly with the endothelium or subendothelial matrix or indirectly via other blood proteins and/or cells, including platelets and neutrophils, which affect platelet biophysically and biochemically, support thrombin generation, and importantly to interact with platelets, endothelial cells, and fibrinogen [47,48,49,50]. Our observation that nucleated cells and their contents specifically tend to accumulate at the borders between RBC- and platelet-rich areas further points to a more specific role RBCs might be playing during early thrombosis. Among the other non-cellular components, we identified an abundance of VWF in all thrombi. VWF was mainly seen in the platelet-rich areas where it was associated with dense fibrin networks and infiltrated platelets. RBC-rich areas, on the other hand, were almost devoid of any VWF. VWF is the main ligand of the glycoprotein Ib platelet-subunit alpha GPIbα (CD42b) [8], mediating the first critical step in platelet adhesion [51,52]. In the subsequent events that culminate in the activation of platelets, endogenous VWF released from the platelet α-granule together with adsorbed surface VWF initiate platelet recruitment that is key to normal thrombus development [53]. Thus, interfering with this interaction by GPIbα-VWF blockade can influence thrombin formation. Indeed, blockade of the VWF- and GPIbα-mediated platelet adhesion resulted in thrombus dissolution and vessel Patency in animal models [54,55]. In a related study, an alternate strategy of rendering VWF less functional by proteolytic cleavage was adopted. VWF cleaving ADAMTS13 protease [56,57] decreased thrombus formation in mice [23,25,26], or alternatively a Adamts13 -/- mice deficient in ADAMTS13 demonstrated faster occlusive MCA thrombus formation [24]. Despite the caveat that a limited number of thrombi samples were available for this study, an inverse relation between red blood cell content and VWF was identified, in line with the study by Denorme F et al. [24]. Hence, the interpretation that ADAMTS13-based thrombolysis can be adopted for patients after determining the thrombus composition from radiological signs on admission [58,59] is further supported by our study.

Apart from the non-nucleated cellular components such as platelets, we also identified a large population of nucleated cells, mainly in the platelet-rich areas of the retrieved thrombi. Immunohistochemical examination showed that these cells are leukocytes. The importance of leukocytes in thrombus formation has been elegantly demonstrated by earlier studies [46,58,60,61]. Neutrophils are the first cells present at the thrombosis site following laser beam injury [62]. In addition to their role in localizing the procoagulant factors such as tissue factor and factor XII [62,63], leukocytes in particular neutrophils play an important role in thrombosis by releasing meshwork of decondensed DNA filaments, referred to as neutrophil extracellular traps (NETs) [64] into the extracellular space.

NETs were initially reported to be released in response to infections by activated leukocytes such as neutrophils, eosinophils, and mast cells [19,38,65,66,67]. Apart from the decondensed chromatin, NETs also contain antimicrobial granule proteins that mainly serve antimicrobial functions [68]. The NET formation can be triggered by multiple signals involving several signaling mechanisms. The extruded NETs entrap and immobilize microbes in areas of locally retained lethal concentrations of effector proteins including proteases for effective bacterial killing [19,69]. This way, NETs can effectively neutralize infection and form an important constituent of host defense. However, recent studies have shown that NETs provide additional scaffold and stimulus for thrombus formation by triggering platelet adhesion, activation, and aggregation [38]. NETs recruit RBCs, and prothrombotic molecules and promote fibrin deposition [38,70,71], and when dismantled by DNase or the anticoagulant heparin, thrombus formation is prevented [38].

Over the years, multiple animal models of thrombosis have been generated that beautifully reproduce NET formation [72]. However, only a few studies have reported NET formation in thrombi from acute stroke patients [17,18]. We identified NETs as an important constituent of thrombi retrieved from AIS patients as detected by the presence of intracellular decondensed nuclear material inside neutrophils, and extracellularly as diffused DNA decorated with citrullinated histones. Importantly, retrieved thrombi showed NETs existing in close association with VWF and platelets at the border areas between RBC-rich and platelet-rich, and the thrombi periphery. NETs are known to render fibrin less susceptible to t-PA-mediated proteolytic degradation [73]. Similarly, t-PA-mediated thrombolysis is more effective when combined with DNAse-1 in ex vivo thrombolysis experiments [17,18]. Importantly, inhibiting NETosis using a NET-inhibitory peptide (nNIF) induced broad-spectrum protective effects after stroke in a mouse model [27]. Therefore, it is not surprising that the patient cohort in this study showed high t-PA resistance.

VWF-mediated initial platelet-activation and aggregation [74] and the subsequent crosstalk between platelets and neutrophils are well-known to lead to the NET formation [75,76,77]. This cross-talk is mediated by several mechanisms involving surface receptors such as cell-adhesion molecules [78] or soluble mediators secreted by activated platelets such as HMGB1 [40]. Platelet-derived HMGB1 mediates the platelet–neutrophil intercellular signaling via the receptor for advanced glycation end products [40,79]. This triggers mixed lineage kinase-like (MLKL) phosphorylation and its activation, which in turn contributes to venous thrombosis by driving NET release from the neutrophils [80]. In a recent study, elevated levels of platelet–neutrophil aggregates along with elevated plasma and platelet surface-expressed HMGB1 levels were observed in stroke patients [27]. The same study utilized isolated platelets from healthy donors and platelet-specific HMGB1-knockout mice to demonstrate platelet-specific HMGB1 involvement in NET formation. Further, platelet-specific HMGB1-knockout mice not only had reduced NET formation after stroke induction but also improved stroke outcomes [27]. In line with these observations, a close association between platelets (visualized by GPIbα and HMGB1) and neutrophils (visualized by myeloperoxidase and neutrophil elastase) in areas rich in NETs was observed. The caveat is that in our study, the source of HMGB1, whether it is platelet- or neutrophil-specific, could not be verified in the thrombi specimens. Despite this limitation, this close association between platelets and neutrophils aided by HMGB1 in our AIS patient thrombi samples further emphasizes its role in NET formation during thrombosis.

The findings from this study have relevant clinical implications for acute stroke management. We identified a dense fibrin network along with VWF and NETs in patient thrombi, known to provide a scaffold for red blood cells, platelets, fibrin, and coagulation factors to adhere to, and render thrombi less susceptible to dissolution. In conclusion, our study supports the notion of a clot dissolution therapy targeting multiple rather than a single component for a successful pharmacological thrombolytic therapy.

## 4. Materials and Methods

The study adhered to the tenets of the Declaration of Helsinki and was approved by the Institutional Review Board and informed consent was obtained. The data were prospectively collected at a tertiary referral center with a well-established comprehensive stroke service accredited by Joint Commission International. The stroke service includes acute stroke diagnostic, stroke units, vascular interventional services, vascular neurological surgery, and rehabilitation services. An acute stroke team provides a rapid assessment service 24 h a day, 7 days a week. All acute stroke patients with large vessel occlusion and eligible for thrombolysis and thrombectomy or thrombectomy alone were included in the study from July 2019 to January 2020. The study was terminated prematurely due to the COVID-19 pandemic.

### 4.1. Thrombi Histology

Thrombi were retrieved, removed gently from the catheter, saline-washed, immediately fixed in 4% paraformaldehyde for 24 h at room temperature, and dehydrated by sequential incubation in a concentration series of ethanol and chloroform. Next, the fixed thrombi samples were prepared by paraffin (Leica, Paraplast, Leider Lane, Buffalo Grove, IL, USA) embedding, and 4–6 µm sections were obtained from each paraffin-embedded thrombus sample using a microtome (Leica RM2255). On average, three sections (top, mid, and bottom layer) from each thrombus sample were analyzed to check the homogeneity of the thrombi samples for the contents viz a viz fibrin, RBCs, and platelets. Different sections from a single thrombus did not differ significantly in the general organization and quantity of these contents (Supplementary Figure S6). Hence, one representative section per thrombus, covering a large thrombus surface, was chosen for the subsequent studies.

Subsequently, thrombi sections were stained with hematoxylin and eosin (H&E; MHS16, HT110116, Sigma Aldrich, St. Louis, MO, USA) to distinguish the erythrocyte- (red) and platelet-rich (pink) areas. Additionally, Martius Scarlet Blue (MSB; RRSK2-100, Atom Scientific Ltd., Hyde, Cheshire, SK144GX, UK) was performed to determine the red blood cell (yellow) and fibrin-rich (dark pink/red) areas. For immunohistochemistry, sections were deparaffinized by heating the slides to 55 °C followed by xylene washes and rehydration using graded concentrations of ethanol in water and pure water at the end. For antigen retrieval, slides were sub-boiled in sodium citrate (10 mM, pH 6) buffer in a microwave oven for efficient epitope exposure to the antibodies, except for VWF and platelet staining. Endogenous peroxidase was quenched by treating the deparaffinized sections with 3% H_2_O_2_, and nonspecific binding of antibodies was eliminated by applying 5% serum (goat or donkey) and 3% BSA for 1 h at room temperature. The primary antibody prepared in 5% serum (goat/donkey) was applied and slides incubated overnight at 4 °C, followed by incubation in respective secondary antibodies for 30 min at room temperature in a humidified chamber. For sera and antibody dilutions, SignalStain Antibody Diluent (#8112, Cell Signaling Technology, Danvers, MA, USA) was used, and the washing steps were performed in 1X Tris Buffered Saline (TBS; 1706435, Biorad, 1000 Alfred Nobel Drive, Hercules, CA, USA) with Triton X-100 (MKBW1852V, Sigma Aldrich, 3050 Spruce Street, St. Louis, MO, USA). For the Secondary Antibody Detection System and Substrate, we used SignalStain Boost IHC Detection Reagents (HRP Mouse #8125; HRP Rabbit #8114) and SignalStain DAB Substrate Kit (#8059), respectively. Nuclei were counterstained with hematoxylin (RRSP72-A and RRSP72-B, Atom Scientific, Hyde, Cheshire, SK144GX, UK) when desired, and slides dehydrated, cleared with 2 changes in xylene (1 min each), and mounted using a xylene-based mounting medium (CureMount, #62804-02, Electron Microscopy Sciences, 1560 Industry Road, Hatfield, PA 19440, USA). The following antibodies were used: rabbit anti-human VWF polyclonal antibody (1:1500, A008202-2, Dako, Glostrup, Denmark), rabbit anti-human fibrin(ogen) polyclonal antibody (1:1000, A0080, Dako), GPIbα monoclonal antibody for platelets (1:100, MA5-11642, Invitrogen, Waltham, MA, USA), mouse anti-human CD45 monoclonal antibody (1:1000, 304002, Biolegend, San Diego, CA, USA) for leukocytes, and rabbit anti-human histone H3 (citrulline R2 + 8 + 17, H3Cit) polyclonal antibody (0.3 µg/mL, ab5103; Abcam, Cambridge, UK). Images were captured with a Nikon Eclipse Ni and AXIO Imager M2 (Zeiss, Oberkochen, Germany). For omission controls, primary antibodies were omitted by incubating a section with the diluent of the respective antibody (1% BSA, 1X Tris Buffered Saline with Triton X-100) to nullify any nonspecific signal from the detection system. For isotype controls, sections were incubated with the respective isotype of the primary antibody in 1% BSA, 1X Tris Buffered Saline with Triton X-100 to nullify any nonspecific binding of the primary antibodies to the thrombi tissue (Supplementary Figure S7). Isotype antibodies used were polyclonal rabbit IgG isotype control antibody (910801; Biolegend, San Diego, CA 92121, USA) or monoclonal mouse IgG1κ isotype control antibody (401402, Biolegend).

### 4.2. Immunofluorescence Staining

For Immunofluorescence, deparaffinized thrombi sections after antigen retrieval (using sodium citrate 10 mM, pH 6 as described above) were blocked with 5% serum (goat and/or donkey, depending on the secondary antibody combinations) and 3% BSA in SignalStain Antibody Diluent (#8112) for 1 h at room temperature and incubated in primary antibodies overnight at 4 °C. The next day, the slides were washed 3 times for 5 min in TBST and incubated with the corresponding secondary antibodies (1.5 µg/mL) for 1 h at RT in a humidified chamber. Thereafter, the slides were washed 3 times for 5 min in TBST, dehydrated in increasing concentrations of ethanol, followed by a quick wash in xylene. Finally, the slides were mounted using Prolong Gold Antifade Mountant with DAPI (4,6-diamidino-2-phenylindole; P36935, Invitrogen, Waltham, MA, USA) as a counterstain for DNA. The following primary antibody combinations were used: rabbit anti-human fibrin(ogen) polyclonal antibody recognizing both fibrin and fibrinogen (1:1000, A0080, Dako, DK-2600 Glostrup, Denmark), sheep anti-human VWF polyclonal antibody (1:250, ab11713, Abcam, Cambridge, UK), rabbit anti-human histone H3 (citrulline R2 + 8 + 17) polyclonal antibody (0.3 μg/mL, ab5103), goat anti-MPO (2 µg/mL; AF3667, R&D Systems, 614 McKinkey Place NE, Minneapolis, MN, USA), mouse anti-hELA2 (2 µg/mL; MAB91671, R&D Systems), mouse anti-human GPIbα monoclonal antibody (1:100, MA5-11642, Invitrogen, Waltham, MA, USA), and/or rabbit anti-HMGB1 (2 µg/mL; ab18256, ABCAM, Cambridge, UK) in 3% normal serum (donkey and/or goat), 3% BSA, and 0.1% Triton X-100 in TBS. Corresponding secondary antibodies used were: Alexa Fluor 488 donkey anti-rabbit IgG (2 µg/mL, A21206; Invitrogen), Alexa Fluor Plus 555 goat anti-mouse IgG (2 µg/mL, A32727, Invitrogen), donkey anti-goat AF546 (2 µg/mL; a-11056), Thermo Fisher Scientific, 168 Third Avenue, Waltham, MA, USA), donkey anti-mouse AF647 (2 µg/mL; A-31571, Thermo Fisher Scientific), and Alexa Fluor 647 donkey anti-sheep IgG (2 µg/mL, A21488, Invitrogen) in normal serum (donkey and/or goat), 3% BSA, and 0.1% Triton X-100 in TBS.

For omission controls, primary antibodies were omitted by incubating a section with the diluent of the respective antibody (1% BSA, 1X Tris Buffered Saline with Triton X-100) to nullify any nonspecific signal from the detection system. For isotype controls, sections were incubated with the respective isotype of the primary antibody in 1% BSA, 1X Tris Buffered Saline with Triton X-100 (polyclonal rabbit IgG isotype control antibody (910801; Biolegend), a polyclonal sheep IgG isotype control antibody (31243, Invitrogen), a monoclonal mouse IgG1κ isotype control antibody (401402, Biolegend) or the respective combination of the isotype primary antibodies to nullify any nonspecific binding of the primary antibodies to the thrombi tissue (Supplementary Figure S8). Red blood cells owing to their inherent autofluorescence were visualized at 555 nm. Axio Imager M2 fluorescent microscope (Zeiss, Oberkochen, Germany) was used for immunofluorescence imaging and images processed by Zen 2012 (blue edition, version 2.3, Zeiss) software.

### 4.3. Hematoxylin and Eosin

Deparaffinized thrombi sections were rehydrated in graded concentrations of ethanol in water and pure water at the end as described above. Slides were stained in hematoxylin solution for 8 min, washed in running tap water for 5 min, followed by differentiation in 1% acid alcohol for 30 s. After another quick wash in running tap water for 1 min, slides were immersed in Scott’s tap water (S5134-100 ML, Sigma Aldrich, Hannover, Germany) for 1 min and washed well in running water for 5 min, followed by rinsing in 95% alcohol (30 s). Slides were counterstained in eosin-phloxine B solution (or eosin Y solution, HT110116, Sigma Aldrich) for 1 min and dehydrated through 95% alcohol, 2 changes in absolute alcohol, 5 min each. Slides were finally cleared with 2 changes in xylene (5 min each) and mounted with a xylene-based mounting medium (CureMount, #62804-02, Electron Microscopy Sciences). Basophilic components such as nuclei are stained purple in hematoxylin, whereas acidophilic components such as fibrin/platelet aggregates are stained pink, and red blood cells red in eosin.

### 4.4. Martius Scarlet Blue

To differentiate collagen (blue), fibrin (red), muscle (pale red), and red blood cells (yellow), the trichrome technique using Martius Scarlet Blue (MSB) staining was used as per the standard procedures (MSB, RRSK2-100). Briefly, dewaxed sections after dehydration through alcohols were stained for nuclei with Weigert’s Iron hemotoxylin supplied with the kit for 10 min, after which the sections were washed quickly in distilled water and differentiated in 1% acid alcohol solution for 30 s. The sections were thereafter washed in running tap water for 3 min, died blue in Scott’s tap water (S5134-100 ML, Sigma Aldrich, Hannover, Germany) for 1 min, and washed well in running water for 5 min followed by rinsing in 95% alcohol (30 s). Next, sections were stained with Martius yellow solution for 5 min, washed quickly in running tap water for 30 s followed by staining with the crystal scarlet solution for 5 min. Subsequently, sections were washed in running tap water for 5 min and treated with a phosphotungstic acid solution for 10 min. After another round of washing in running tap water for 5 min, sections were stained with aniline blue solution for 5 min, washed in tap water for 5 min, followed by rapid dehydration through solutions of increasing ethanol concentration until 100% ethanol was reached. Finally, sections were immersed in xylene and mounted using CureMount (#62804-02, Electron Microscopy Sciences). All the steps were carried out at room temperature.

### 4.5. Quantification of RBC-Rich and Platelet-Rich Area

To quantify the RBC-rich and platelet-rich areas, we followed the methods described by Staessens et al. [42], with few modifications. Briefly, MSB staining of at least 3 sections from each thrombus representing the top-most, middle-, and bottom-most layer was used to quantify RBC-rich and platelet-rich areas. Individual thrombus images from each section were exported in a 5× resolution and stitched using ImageJ software 1.49 v (National Institutes of Health, Bethesda, MD, USA; http://imagej.nig.gov/ij/ (accessed on 15 March 2021) ). To prevent a false positive signal in the quantification, the background from the stitched images surrounding the thrombus section was removed using Photopea (https://www.photopea.com/ (accessed on 20 March 2021)). Color segmentation and planimetric analysis of the MSB stainings were performed using ImageJ software 1.49 v (National Institutes of Health, Bethesda, MD, USA; http://imagej.nig.gov/ij/ (accessed on 5 April 2021) to calculate the percentage of RBC-rich area (yellow) of the total section area. The remaining areas (pink to red) were platelet-rich and calculated as 100% minus the determined % age of the RBC-rich area. Based on their abundance, thrombi were classified as platelet-rich (total RBC-rich area < 15%), mixed (total RBC-rich area 15–30%), or RBC-rich (total RBC-rich area > 30%).

### 4.6. Statistical Analysis

Data are presented as mean ± standard error of the mean (SEM) unless mentioned otherwise. To test for significant differences between groups, *t*-tests with post-hoc Bonferroni adjustment were used where *p* < 0.05 were considered significant. GraphPad Prism software (version 7.0a, San Diego, CA, USA) was used to perform the statistical analysis.

## 5. Conclusions

Our study identified an extensive network of fibrin, VWF, and NETs in the thrombi retrieved from acute ischemic stroke (AIS) patients after undergoing mechanical thrombectomy at Hamad Medical Corporation, Doha. We identified a close association between thrombi components specific to platelets and neutrophils known to mediate NET formation in neutrophils and providing a scaffold for thrombus stability. These findings underline the need to consider an amalgam of strategies targeting multiple scaffolding components for an efficient pharmacological recanalization, as a promising therapeutic approach for ischemic stroke.

## Data Availability

Not applicable.

## Supplementary Materials

The following supporting information can be downloaded at: https://www.mdpi.com/article/10.3390/ijms232214477/s1.

## Abbreviations

ADAMTS13: a disintegrin and metalloproteinase with thrombospondin motifs; AIS: acute ischemic stroke, GPIbα: glycoprotein Ib platelet-subunit alpha; H and E: hematoxylin and eosin, HMGB1: platelet-derived high-mobility group box 1, MCA: middle cerebral artery; MLKL: mixed lineage kinase-like; MPO: myeloperoxidase; MSB: Martius scarlet blue; NE: neutrophil elastase; NETs: neutrophil extracellular traps; RBCs: red blood cells; tPA: tissue plasminogen activator; VWF: von Willebrand factor.
