# Supplementary material for: Platelet–Neutrophil Association in NETs-Rich Areas in the Retrieved AIS Patient Thrombi
Source: Int J Mol Sci. 2022 Nov 21;23(22):14477. doi: 10.3390/ijms232214477 (PMC9694992; doi:10.3390/ijms232214477)
Supplement: Supplementary file 1 [file ijms-23-14477-s001.zip › ijms-1980582-supplementary.pdf]

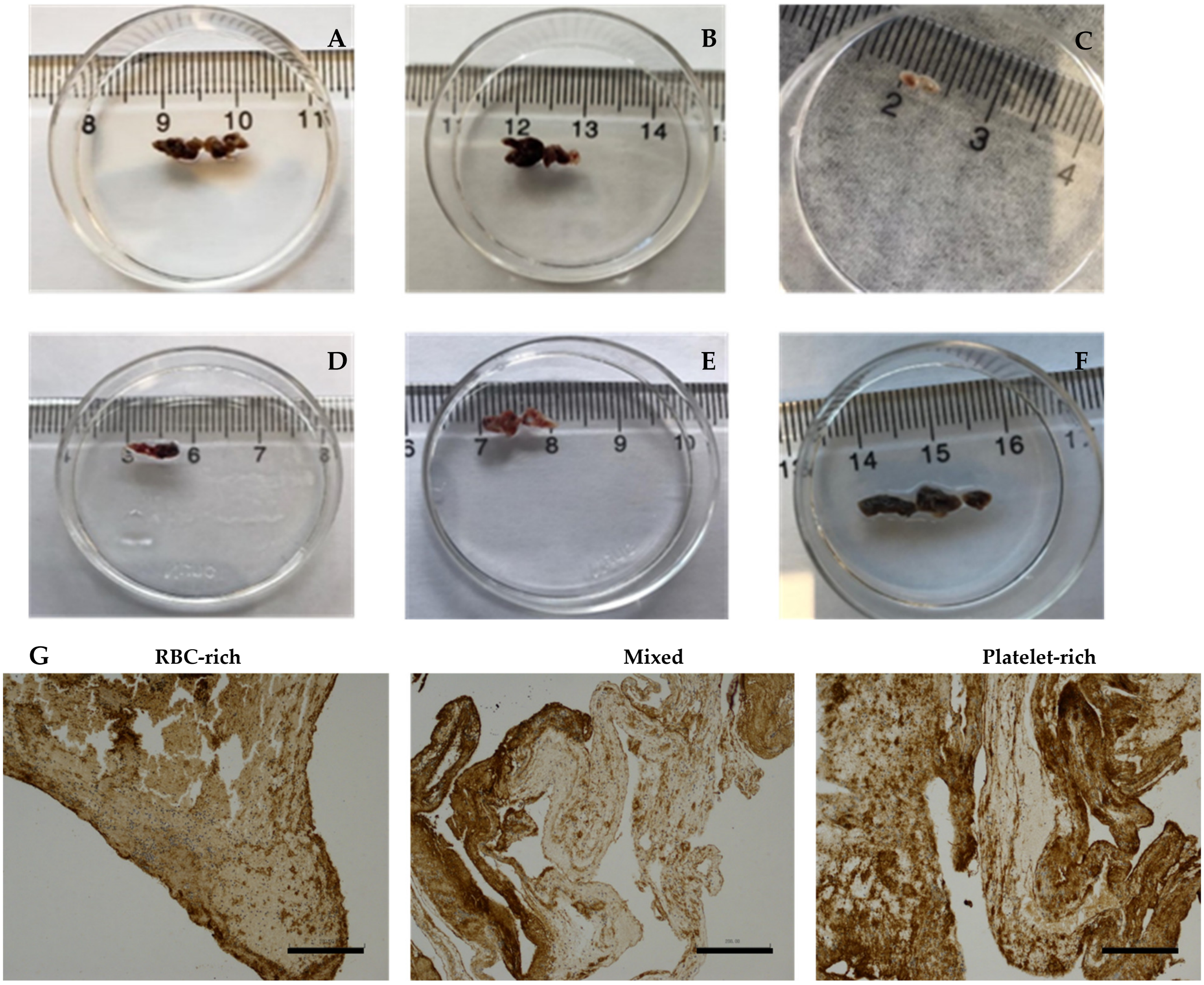

Supplemental Figure S1. Retrieved thrombi from AIS patients. (Thrombi were gently removed from the stent retriever after thrombectomy and pictured to show the gross view of the thrombus material.

Representative thrombi (A-F) from 6 patients show their varying shape, size, and color (scale = cm). (G) Representative immunohistochemical image of RBC-rich, mixed, and platelet-rich clot stained for platelets using GPIbα antibody. Platelet-rich clot shows the most intensive staining compared to the other two. Irrespective of the class, stronger staining is seen in the platelet-rich areas and the outer edges. Scale bars: 200 μm.

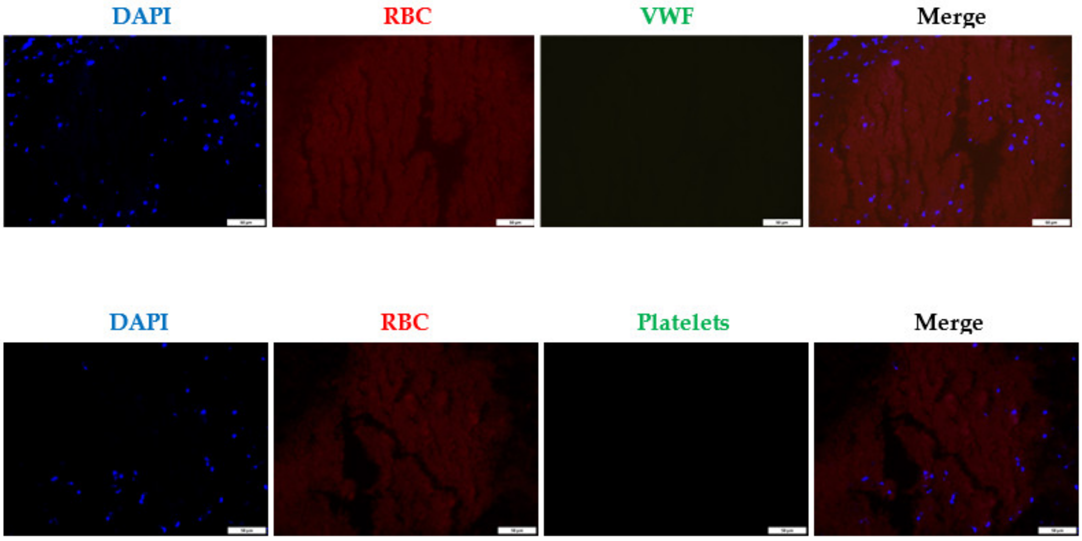

Supplementary Figure S2.Composition of the RBC-rich areas in stroke thrombi. Representative immunofluorescence image of stroke thrombi stained for DNA (DAPI), and with antibody against VWF, RBCs are apparent by their autofluorescence at 555 nm (upper panel). Note the RBC-rich area devoid of any VWF, and contains very few nucleated cells. Scale bar = 50 μm. Representative immunofluorescence image of stroke thrombi stained for DNA (DAPI), and with antibody against platelets (GPIbα) (lower panel). Note the RBC-rich area contains very few nucleated cells and is devoid of any platelets. Scale bar = 50 μm.

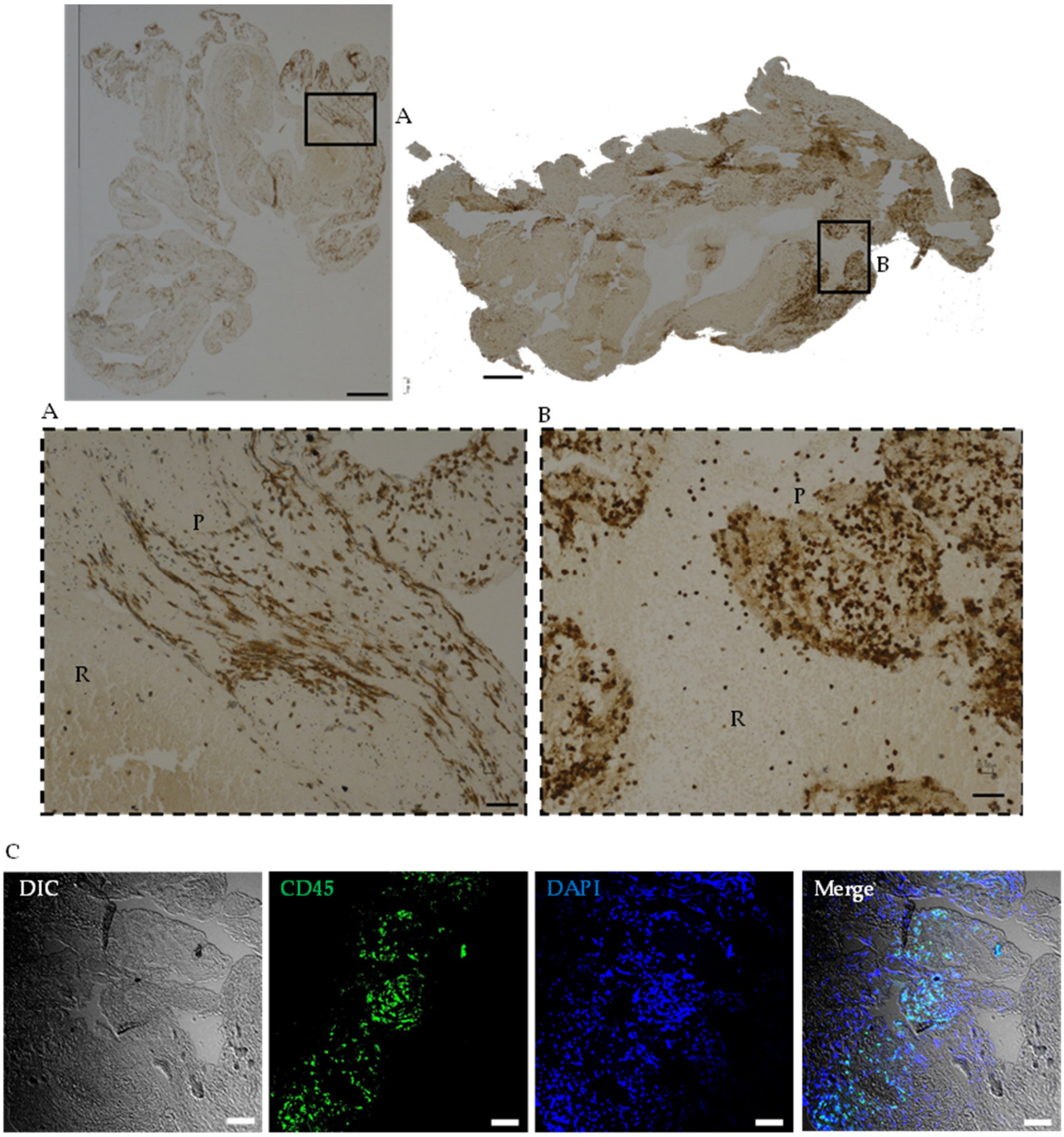

Supplementary Figure S3. Abundant leukocyte accumulation mainly at the interface between platelet-rich and RBC-rich areas. (A-B) Representative immunohistochemical staining of platelet-rich (left, upper panel) and RBC-rich (right, upper panel) thrombi stained for leukocytes using CD45 antibody. Higher magnification of the representative stainings is shown in inserts (middl– panels - A, B). Note the RBC-ri©areas (R) containing very few leukocytes. Scale bars: 500 µm (top panel) and 50 µm (low©panels). (C) Representative image of stroke thrombus stained with DNA (blue, DAPI) and CD45 antibody specific for leukocytes. Scale bars are 100 µm.

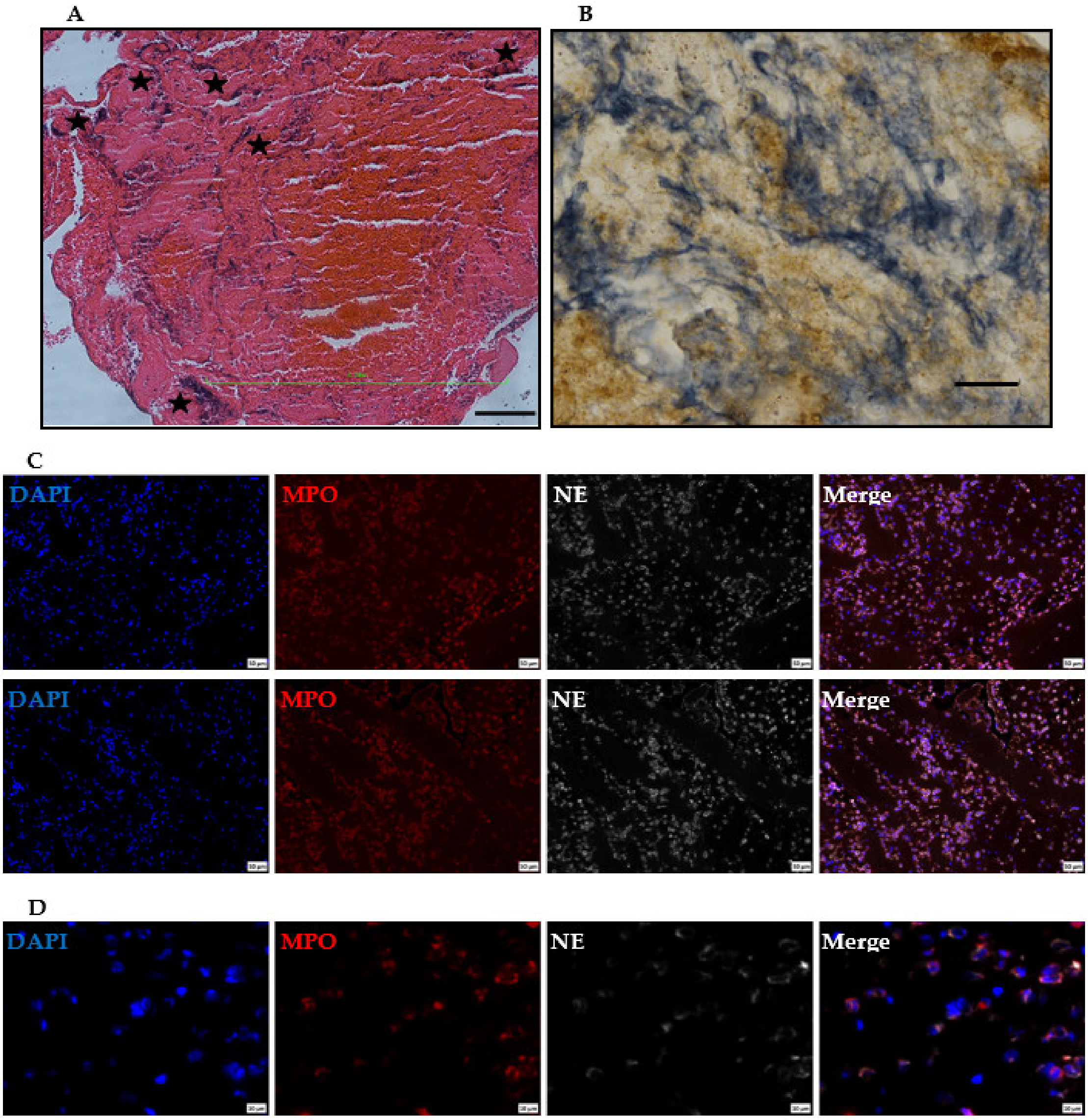

Supplementary Figure S4. Platelet-rich areas show diffused nuclear material and abundant neutrophils. (A) Representative H&E stained image of a thrombus depicting highly populated polymorphonuclear cells and extracellular nucleic acid (*) predominantly located in the outer layer of the thrombus. Scale bar= 20µm. (B) Representative histochemical image of stroke thrombus. Note the diffused DNA fibrous material stained blue with hematoxylin. Scale bar = 20µm. (C) Representative immunofluorescence images of ischemic stroke patient thrombi stained with antibodies against neutrophil-specific markers myeloperoxidase (MPO) and neutrophil elastase (NE) and DNA (DAPI). Scale bar = 50µm. (D) A magnified image showing neutrophils double-stained with MPO and NE, and DAPI (DNA). Scale bar 10µm.

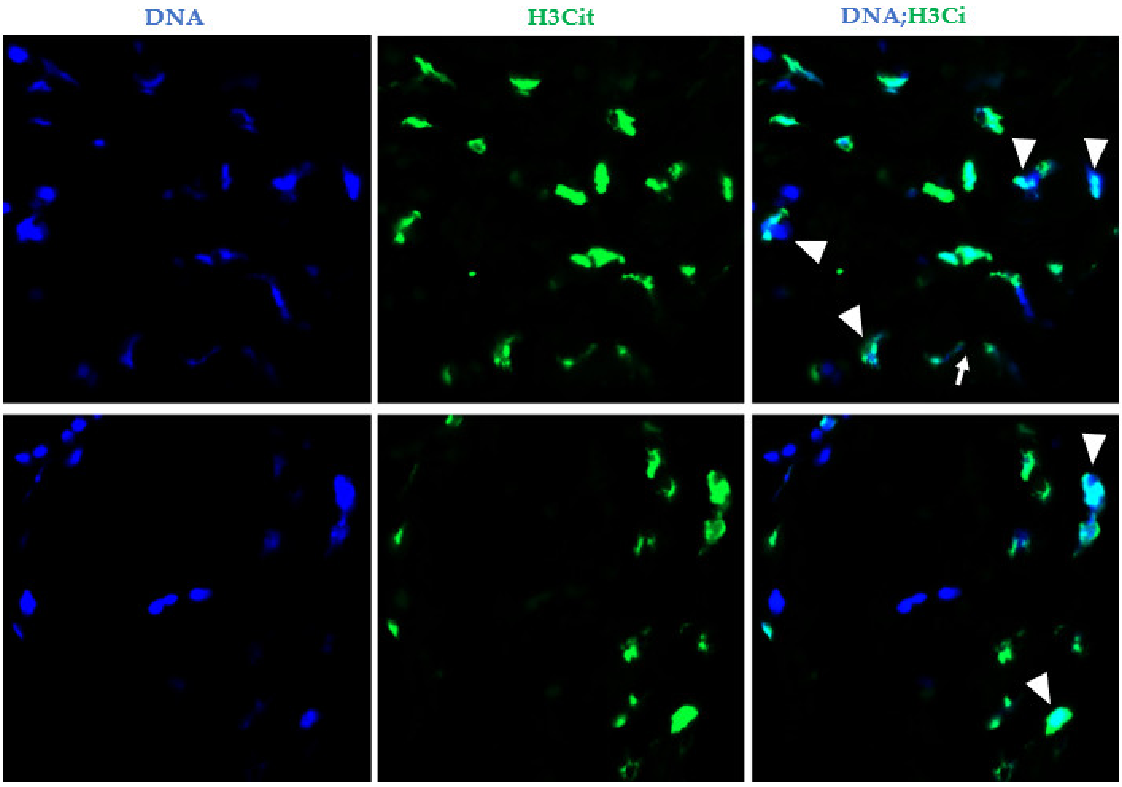

Supplementary Figure S5. Stroke thrombi show NET formation. Human stroke thrombi sections were stained for DNA (DAPI, blue), and with antibodies against citrullinated histone (H3Cit, green) to visualize NET formation. Co-localization of DNA (DAPI, blue) and citrullinated histones (green) points to NET.

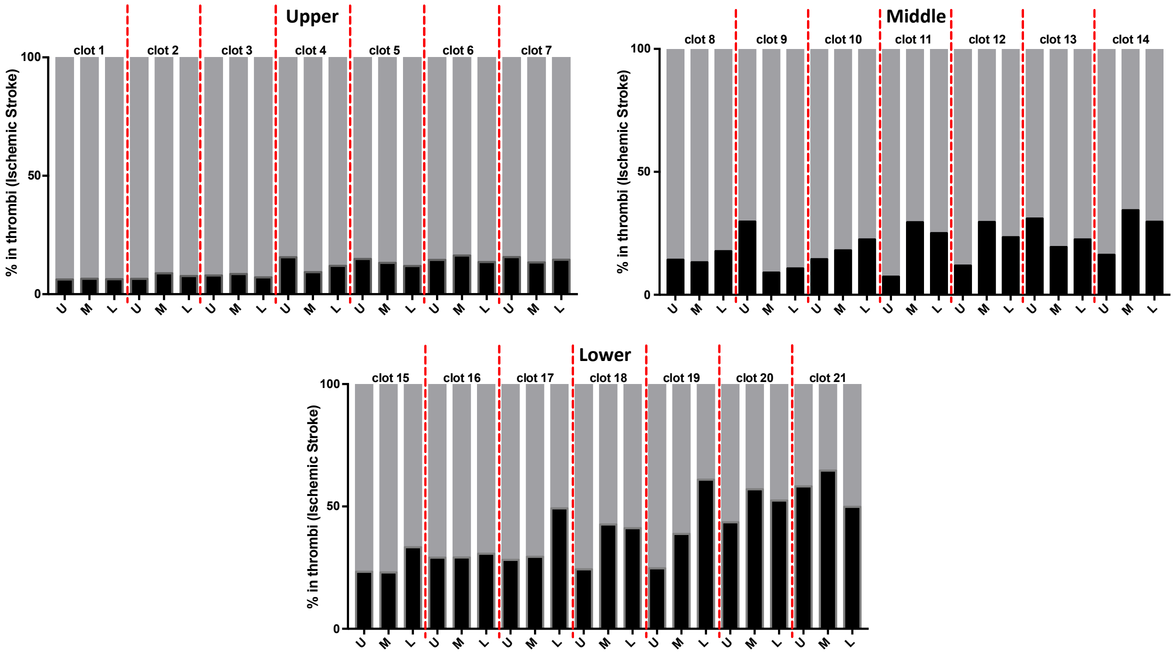

Supplemental Figure S6. Retrieved thrombi from AIS patients. (A) Quantitative analysis of the stroke thrombi sections representing upper-, middle- and the lower-layer using H&E staining was performed and the mean percentage of RBC-rich areas (black) and platelet-rich areas (grey) was determined. All the thrombi contain significant amounts of platelet-rich and RBC-rich areas; however, individual thrombi differ in composition with some showing a high abundance of platelet-rich areas while others high abundance of RBC-rich areas (n=21, vertical bars).

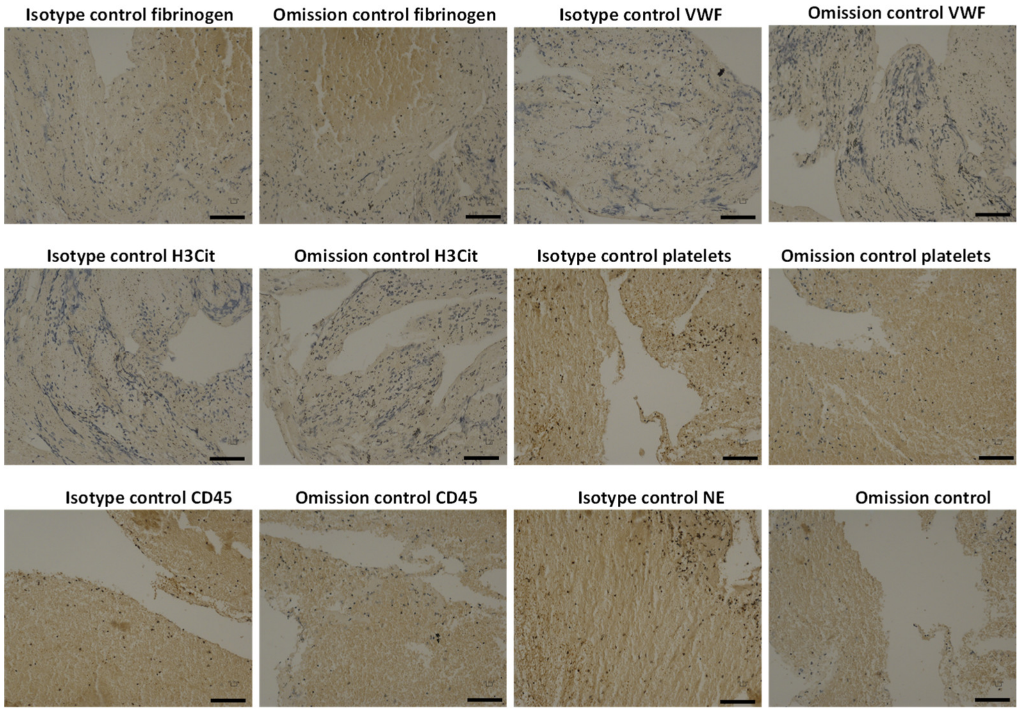

Supplementary Figure S7. Isotype and Omission Controls.(A) Isotype-matched and omission controls for fibrinogen (A), VWF (B), H3Cit (C), platelets (D), CD45 (E), and neutrophil elastase NE (F) lack any positive signal. Scale bars are 100 µm.

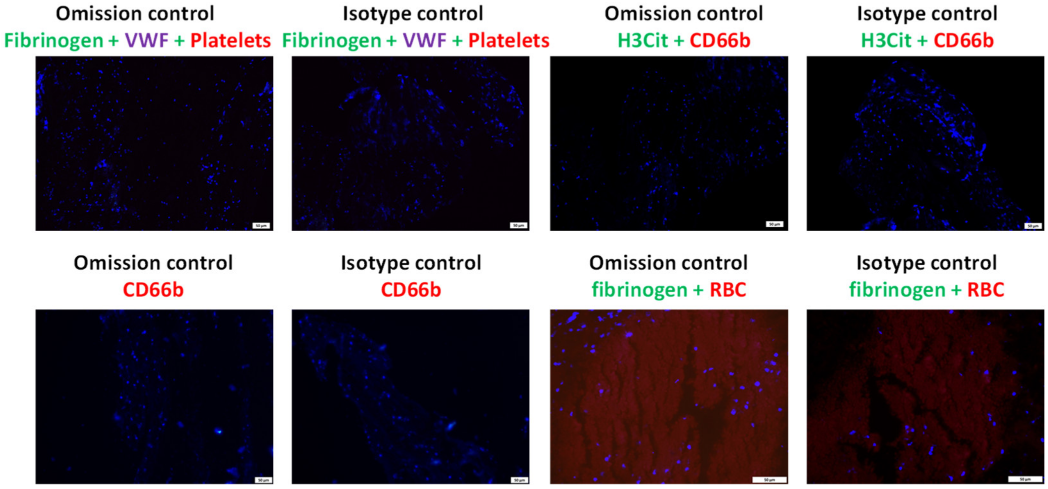

Supplementary Figure S8. Controls. Omission and isotype controls of the fluorescent costainings. No positive signal is detected for fibrin (green), VWF (violet), platelets (red), and CD66b (red). Scale bars are 50 µm.

**Supplemental Table S1. Patient characteristics**	
**Age in years, mean (±SD)**	51 (± 11)
**Sex, (male) n (%)**	17 (81)
**Ethnicity, n (%)**	
Arab	4 (19)
South Asian	11 (52)
Far Eastern	1 (5)
African	5 (24)
**Location thrombus, n (%)**	
Middle Cerebral Artery (MCA)	19 (90)
Posterior Cerebral Artery (PCA)	2 (10)
**Cardiovascular risk factors, n (%)**	
Current smoking	5 (24)
Hypertension	11 (52)
Diabetes Mellitus	10 (47)
Dyslipidemia	6 (28)
Atrial Fibrillation	3 (14)
Chronic Anticoagulation use, n (%)	1 (5)
Anti-platelet Therapy use, n (%)	2 (10)
**Baseline NIHSS, median (range)**	15 (6 – 24)
**Onset time to CT in minutes, median (range)**	130 (51 – 480)

SD: standard deviation, NIHSS: National Institutes of Health Stroke Scale, CT: Computed Tomography.
